# Therapy without faith: Muslim clients' experience of religious exclusion and minimisation in therapy

**DOI:** 10.1111/papt.70019

**Published:** 2025-10-24

**Authors:** Rumena Islam, Paul Chadwick

**Affiliations:** ^1^ Doctoral College/Department of Psychology University of Bath Bath UK

**Keywords:** client experiences, cultural sensitivity, Islam, mental health, religion, thematic analysis

## Abstract

**Objective:**

The integration of religious beliefs is considered an essential component of evidence‐based practice; however, clients from faith‐based communities frequently report that their beliefs are overlooked in therapy. While existing research primarily centres on therapists' perspectives, there is limited understanding of how Muslim clients themselves make sense of therapy when their religious identity is not acknowledged, particularly within mental health systems often grounded in Western psychological models. This study, therefore, aimed to explore the mental healthcare experiences of Muslim therapy users in the UK who received therapy where their religion was perceived as minimised or excluded, despite its personal significance.

**Design:**

A qualitative approach was adopted, underpinned by a critical realist epistemology. Semi‐structured interviews were conducted with 25 Muslim participants, aged 18–56 years. These interviews were conducted remotely via Teams and subsequently transcribed for analysis. Data analysis was performed using reflexive thematic analysis.

**Results:**

Three overarching themes were generated through the analysis: feeling powerless and unseen when attempting to bring Islam, shaking my foundation and feeling compelled to choose between my faith or therapy. Participants described a significant emotional impact when Islam was excluded or minimised.

**Conclusion:**

These findings highlight the need for therapists to actively explore and integrate clients' religious worldviews, and for services to consider culturally responsive practices. The findings also suggest a need for enhanced cultural competence training and systemic changes to improve the therapeutic experience for Muslim clients. This study offers insight for developing more inclusive and effective mental health practices as well as implications for clinical practice.

**Practitioner points**

- Muslim clients in this study reported that religion was often excluded or minimised in therapy, leading to feelings of disconnection, self‐censorship and disengagement.
- Integrating religion into mental health services offers opportunities to improve engagement, rapport and outcomes for Muslim clients.
- Therapeutic models that neglect a client's religion may inadvertently invalidate important sources of meaning and coping.

## INTRODUCTION

Rates of mental health difficulties in the United Kingdom (UK) have risen significantly in recent years (Wykes et al., 2023). Despite growing public awareness and expanding services within the National Health Service (NHS), substantial disparities persist in who accesses psychological support (Wang et al., 2024; Westberg et al., 2022). Individuals from religiously minoritised communities demonstrate lower utilisation of mental health services and are more likely to report negative experiences when accessing such services (Bignall et al., 2019). Muslims, who constitute the second largest faith group in the population (6.5%) (Office for National Statistics [ONS], 2022), are among those for whom the patterns are particularly evident. Muslims are significantly underrepresented in services like NHS Talking Therapies (Ahmad et al., 2022; Alam et al., 2024). For example, in 2021–2022, only 4.4% of service users identified as Muslim, of which only 2.6% finished their course of treatment (NHS Digital, 2022). This suggests that Muslims may encounter additional barriers in accessing and engaging with mental health treatment.

Research suggests that one reason for this underrepresentation may lie in the lack of religious responsiveness within therapy (Meer & Mir, 2014). Muslims often report concerns about whether therapists will understand or respect their faith, and many express fears that their religious identity will be pathologised or misunderstood (McLaughlin et al., 2022; Meer & Mir, 2014). While several studies have explored the importance of culturally competent therapy, few have directly examined the consequence of excluding religious identity within the therapeutic relationship. This is a significant gap given that many Muslims describe their religion as foundational to their well‐being and meaning making (Ayub & Macaulay, 2023; Causier et al., 2024).

Jabeen and Snowden (2022) explored the experiences of NHS mental health professionals in providing mental health care provision for Muslims in the UK, focusing on whether care can be truly person‐centred without considering religious identity. They analysed seven studies, most connected to NHS settings, identifying key barriers such as staff misunderstandings of religious practices, being ill‐prepared, lacking necessary knowledge and experience to work with Muslim patients and unconscious religious bias. Practitioners also reported avoiding religious discussions for fear of offending or overstepping boundaries, resulting in shortcomings in treatment and care. However, while their findings provide valuable service‐level critique, there remains limited exploration of how such exclusion is personally and psychologically experienced within therapy sessions themselves, particularly through clients' own narrative.

This neglect can also be understood through the current mental health framework in the UK, which tends to operate from predominantly Eurocentric secular models. Mainstream therapeutic models such as cognitive behavioural therapy (CBT), psychodynamic and person‐centred therapies are grounded in western, individualistic assumptions about identity, selfhood and mental health (Adams et al., 2018). Therefore, individuals who hold strong beliefs may choose to refrain from or feel unable to engage with them (Hassan et al., 2024). This can lead to the marginalisation or neglect of religious identity in therapeutic contexts.

Internationally, some Muslim majority countries (e.g., Pakistan, Malaysia and Turkey) have taken steps to integrate Islamic principles into psychological treatment, including faith‐informed CBT and Islamic psychology approaches, and have reported a higher level of engagement and satisfaction (Irfan et al., 2017; Sabki et al., 2019; Turgut & Ekşi, 2020). However, these findings are not directly transferable to Western contexts, where Muslims are often minoritised and where healthcare systems are typically secular. In the UK and other Western countries, pilot studies and small‐scale trials have investigated the adaptation of therapeutic approaches for Muslim individuals (e.g., Mir et al., 2019; Naeem, 2019). These studies have shown promising outcomes, such as increased engagement, improved therapeutic alliance and enhanced treatment effectiveness. Despite a growing evidence base, the integration of such adaptations into mainstream psychological services remains limited, highlighting a gap between research findings and service provision.

### Current study

The underrepresentation of Muslim voices in psychological research contributes to a continued space in our understanding of what it means to provide religiously sensitive care. While other faiths have occasionally reported tensions when religion is dismissed in therapy (Cragun & Friedlander, 2012), Muslim clients may face additional challenges linked to Islamophobia, racism or misunderstandings about Islamic practices. This dual burden places Muslim clients at a unique disadvantage, as the exclusion of Islam in therapy may reinforce broader societal invalidations and exacerbate mistrust towards mental health services.

To the researcher's knowledge, no studies have explored the experiences of Muslim individuals in the UK who accessed psychological therapy and perceived their Islamic faith to be excluded or minimised in treatment, even though their religion held significant personal and psychological relevance. Much of the literature that exists focuses on the general importance of religion in therapy for Muslims, therapist attitudes or models of faith‐sensitive therapy, but there remains a scarcity of qualitative work exploring how Muslim clients themselves experience therapeutic spaces. This is important because (a) the UK has a growing Muslim population (Pillaye, 2020), (b) there is a lack of Muslims seeking support for their mental health (Shakoor et al., 2022) and (c) given the salience of religious identity within the Muslim community, understanding the impact of its exclusion is essential for developing more inclusive therapeutic approaches.

### Research questions

- What effect does perceived therapist minimising or excluding religion from therapy, when it is desired by the client, have on the client's experience of therapy?

## METHOD

This study follows the reporting standard outlined by the Consolidated Criteria for Reporting Qualitative Research (COREQ) guidelines (Tong et al., 2007).

### Ethics

The study received ethical approval from the University of Bath Research Ethics Committee (Ref: 0740‐1196).

### Design

A qualitative approach using semi‐structured interviews was used for this study to gather a rich and meaningful understanding of participants' experience of integrating their religious beliefs.

### Participant eligibility

Given the study's focus on personal experiences, eligibility to participate was based on self‐reported experiences. To ensure participants met the eligibility criteria, they were first screened via a 10‐minute interview. This included open questions to obtain brief qualitative accounts of people's subjective experiences without the limitation of predefined responses.

To ensure the sample is appropriate for addressing the research question, the following inclusion criteria were utilised: (a) currently in therapy or must have received psychological therapy from a mental health care professional and perceive that their therapist was at times minimising or excluding their religion from therapy, (b) must be a Muslim living in the UK, (c) must be over the age of 18, (d) must be an English speaker.

Non‐English speakers were excluded from this study due to the complexities involved in accurately interpreting and analysing qualitative data (Braun & Clarke, 2021a).

### Interview schedule

The interview schedule was developed in collaboration with a Patient and Public Involvement (PPI) group comprising 12 members of the UK Muslim community, including five professionals and seven with lived experience of mental health difficulties. Their input ensured the interview schedule was understandable, relevant to the research question and culturally appropriate. PPI feedback was obtained through a survey disseminated via social media and professional networks. Their feedback highlighted areas where the language could be more culturally sensitive and questions could be reworded to use more lay terms. For example, ‘how does Islam intersect with your mental health experiences’ was changed to ‘how does Islam contribute to your mental health?’

A pilot interview was also conducted with an individual that met the study criteria before proceeding with the main study. Based on this feedback, changes were made to improve the conversational flow of the interview and reduce instances where the questions felt overly rigid or formal.

### Recruitment

Participants were recruited using Muslim organisations, mosques and social media. Interested individuals were asked to contact the researcher directly to express interest, and those who met the inclusion criteria were subsequently provided with further details and consent forms before participation. As a stipend for their time, participants received £10 at the end of the interview. Given the flexible and interpretive nature of reflexive thematic analysis (RTA), no fixed requirements are imposed for sample size (Braun & Clarke, 2021b). Therefore, the recruitment of 25 participants was deemed appropriate for the aims of this study.

### Screening for ‘imposter participants’

In response to the increasing occurrence of ‘imposter participants’ enrolling in online research studies, especially those offering monetary incentives, a systematic protocol was developed to filter out these individuals. This approach was informed by recommendations from Ridge et al. (2023) and Santinele Martino et al. (2024), who stress the importance of early screening to gauge narrative coherence, participant fit and the likelihood of genuine experience. Expressions of interest via email were not followed up if any of the following conditions were met:

- Multiple individuals registered for the study almost simultaneously, just minutes after the advertisement was published.
- Emails from multiple individuals displayed similar formats and often came from the same email service provider.
- Only interested in the payment aspect of the study, rather than in contributing meaningful insights or engaging with the research.
- Emails expressing interest in the study included blank subject lines or consisted of only a few brief sentences. In some cases, potential participants directly copied text from the recruitment advertisements.

Participants who appeared to meet the inclusion criteria were then invited to take part in the 10‐minute screening interview.

### Procedure

All participants received an electronic version of the information sheet and an informed written consent form and were given the chance to ask any questions to the researcher before agreeing to participate. Once consent was obtained and eligibility verified, participants were invited to the interview.

Each participant took part in a semi‐structured interview between February 2024 and June 2024, which lasted between 45 minutes and an hour. This was split, with the first section involving gathering demographic information and the second section exploring the participant's experience of therapy.

All interviews were conducted remotely by the primary researcher (RI) using Microsoft Teams. The platform's ‘record and transcribe’ functions were utilised to produce initial transcripts, which were then reviewed and checked against the recordings for accuracy by the researcher (RI) and research apprentice (EV). At the end of the interview, participants were thanked for their participation and provided with a debrief. Following the verification and anonymisation of transcripts, RTA was conducted.

Consistent with Braun and Clarke's (2021a) RTA, a reflective diary was kept by RI documenting initial thoughts and insights acquired during the research process, including any preconceived biases.

### Data analysis

#### Paradigm and theoretical framework

Data were analysed in accordance with an RTA methodology (Braun & Clarke, 2006), chosen for its flexibility in exploring multifaceted qualitative data. RTA involves identifying, analysing and reporting patterns (themes) within the data, which can provide insight into the experiences and perspectives of the participants whilst also acknowledging the role of the researcher in the process (Braun & Clarke, 2019). The study applied the six‐phase RTA framework set out by Braun and Clarke (2019); see Table S1 for a detailed overview of implementation.

Coding was completed at a semantic and latent level. Inductive coding was used to capture personal, subjective experiences without imposing pre‐existing frameworks or theories on the data and seeing participants as experts on their own experiences (Braun & Clarke, 2021a). However, as noted by Braun and Clarke (2006), a purely inductive approach can run the risk of researcher bias, particularly their epistemological position. The current research included deductive elements—for example, the research question was informed by literature on negative therapy experiences of Muslims. Therefore, it is important to hold and acknowledge both positions when analysing the data (Joffe et al., 2012).

The researcher also took a critical realist ontological stance as it recognises that while Muslim clients' therapeutic experiences are real, their interpretations are shaped by broader social, cultural and religious influences. This approach allows for exploration of the objective reality of therapy, while understanding that clients' insights of how their religious identity is integrated or dismissed are mediated by external societal factors (Braun & Clarke, 2013).

Trustworthiness was rigorously addressed throughout to ensure the integrity and credibility of the findings (Megheirkouni & Moir, 2023). Transparency and traceability of methodological decisions were maintained through a comprehensive summary and audit trail of the analytical journey. Reflexive practices were consistently engaged in, particularly regarding researcher positionality, acknowledging how the primary investigator (RI)'s identity as a Muslim might influence data interpretation and analysis. Credibility was gained through RI's thorough and immersive engagement with the raw data and strengthened by collaborative discussions with other researcher team members, fostering multiple perspectives and challenging assumptions in theme development. Finally, reflexive journaling and peer debriefing were utilised to critically examine researcher biases, assumptions and emotional responses, thereby strengthening the confirmability and overall robustness of the study's conclusions (Ahmed, 2024).

#### Researcher's context

The recruitment and data collection process were carried out by the primary researcher (RI), supported by the research supervisor (PC) and a research apprentice (EV). Personal characteristics of RI to be considered are being in her twenties, British Asian Muslim female and a clinical psychology doctorate student. PC is a White British male, with over 30 years of experience in psychological research and a practicing Clinical Psychologist. EV is a White British female psychology undergraduate who supported the research via a university‐run research apprenticeship scheme, a Turkish British female, an Expert by Experience and a research assistant for a Muslim mental health charity, who reviewed themes and the findings to help mitigate some of the researcher's bias.

## RESULTS

### Participants

Seventeen females and eight males were recruited. Participants' age ranged from 18 to 56, with an average age of 30. Seventeen participants were of British Asian ethnicity, four were Black British and four were Arabs. Of these, seven resided in London, two in Southeast England, six in the Northwest, five in the Northeast, one in the West Midlands, one in the East Midlands, two in Southeast England and one in Wales.

Participants had accessed therapy within the last five years, either through NHS services (*n* = 15), university counselling (*n* = 2) or private practice (*n* = 8). Participants had presented to therapy with a range of psychological difficulties, including anxiety disorder, depression, trauma and psychosis. Some had longstanding mental health conditions, while others were experiencing acute but situational challenges at the time of seeking help.

**FIGURE 1:**
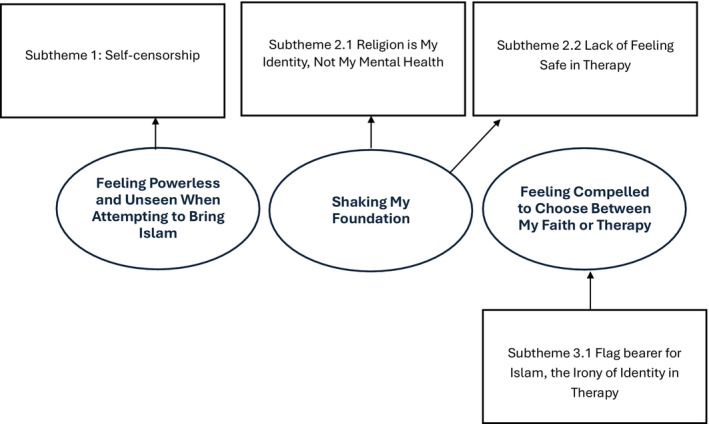
A thematic map depicting final themes, sub‐themes and their relationship. Circles depict themes, rectangles depict sub‐theme.

RTA yielded prominent themes encapsulating the experiences and perspectives of Muslim participants regarding feeling their religion was excluded or minimalised in therapy. Themes were categorised into three key areas informed by the literature and structure of the questioning: feeling powerless and unseen when attempting to bring Islam, shaking my foundation and feeling compelled to choose between my faith or therapy (see Figure 1).

### Theme 1. Feeling powerless and unseen when attempting to bring Islam: ‘I Could Not Bring My Whole Self.’ *(P2)*

This theme lays the foundation for highlighting the disempowerment participants felt when attempting to introduce or integrate Islam into the therapy session. The lack of say over how religious identity was received or acknowledged within the therapeutic space underpins the significance of power imbalance, creating a hierarchy between the client and therapist.

I felt like I was banging my head against the wall when I tried to talk about Islam. I'd be sitting there telling them about what really mattered to me, but it was like they didn't want to hear me. I just felt small like I had no voice in a space that was supposed to be about me. (P18)

Participants described feeling powerless when their attempts to discuss Islam in therapy were met with silence, avoidance, judgement, redirection or misinterpretation. Such responses were often interpreted as a lack of openness, Islamophobia, disinterest or validation, leaving participants feeling that they had no control over what parts of them were seen or taken seriously in therapy. It conveyed a subtle message that religion was peripheral or irrelevant in therapy.

When she didn't respond to anything I said about Islam, it was awkward, so I stopped. I don't think she wanted to talk about Islam. It was like I had no control, I wasn't given the opportunity to focus on what I needed in therapy. (P8)

Other participant voiced that the therapist had an underlying assumption that religion was not important, or worse, a deterrent to effective therapy. This created a sense of helplessness and reflected to Muslim clients that their culturally specific way of conceptualising distress, rooted in faith, was not valid within a therapeutic framework. Participants' experiences highlight that this was not purely a surface‐level issue but linked profoundly to broader feelings of marginalisation and invisibility within mental health services, predominately around their religious identity.

There was this underlying sense that like my faith was problematic or something I had to hide, as if I was not the right type of person the service wanted to treat it made me feel like an outsider. (P4)

### Subtheme 1.1. Self‐censorship

For many, the internal response to this powerlessness was the experience of silencing or censoring their Islamic identity in therapy. While the reason for withholding information varied, all participants communicated a sense of needing to protect parts of themselves. For most, this appeared due to concerns that Islam was either unwelcome, misunderstood, or marginalised within the therapeutic space. This included self‐censoring conversations about religious beliefs, practices and coping strategies, which they considered crucial to their mental health.

I wanted to say I pray five times a day and that was my coping strategy, but because of earlier discomfort of talking about Islam I thought she might think I am extreme in my religion or something. (P5)

The need to filter what they shared was not just a practical choice, but a reflection of the absence of safety and trust they felt in the therapeutic space. This sentiment reveals the broader issue of participants feeling like they had to conform to the therapist's expectations or the perceived confines of what was acceptable to discuss, rather than freely sharing their authentic experiences.

You know when I mentioned Islam, she just rolled her eyes and changed the subject… After that, I just stopped, there was no point. (P13)

For several participants, this dynamic of self‐censoring extended beyond the therapy sessions and impacted the overall experience of mental health care. The magnitude of this suppression was profound; by having to edit their narratives, participants felt a conflict between their true selves and the persona they presented in therapy.

When my therapist made me feel I couldn't talk about Islam I found myself saying what I thought she wanted. It made me realise that mental health support was not made for Muslims like me. (P17)

This impacted the therapeutic relationship, resulting in participants becoming emotionally closed off and creating distance from the therapist, which in turn restricted participants' ability to fully engage and bring their whole selves into therapy.

It just felt like there is some things that I couldn't tell her, and I had to put on a show for her, to pretend she understood me, but really I was just hiding parts of me away […] It was more hard work coming to therapy because I had to also censor myself to fit their narrative. It left me feeling more alone. (P3)

### Theme 2. Shaking my foundation: ‘Don't make me look at my religion like that. I don't like it.’ (P1)

Theme two demonstrates that, when religion was brought into therapy, it was often pathologised rather than validated, producing experiences of destabilisation and compromising participants' sense of safety. Some participants felt their faith was often questioned or treated as separate rather than as part of their identity and recovery.

It was like my therapist was asking me to put my faith to the side before I even sat down. But how do you tell a Muslim to switch Islam off. (P6)

The consequence of this for some is that it led to a sense of rupture not only from the therapy process, but themselves, as they struggled to merge the conflicting messages they were receiving about who they were and how they should manage their mental health difficulties.

That was something she put in my head and that rubbed me up the wrong way because I was like, don't make me look at my religion like that. I don't like it. I didn't like it. I don't like the idea of even looking at religion like that, as that makes me feel guilty. That's a negative feeling. I shouldn't feel these guilts […] I don't want you to question my religion […] And then it was after that, I almost felt like. I had to hold back from her like I was, like calculating my answers, leaving religion out a little bit. (P1)

The above extract highlights how for some the foundation of their identity was shaken, leaving them questioning whether religion was part of the problem rather than the solution. This tension between how some participants viewed their faith and how the therapist positioned it led to many participants to feel conflicted about their identity. For some, therapy became less about getting support for their mental health and more about defending who they were at their core. For others, the process of having their religious identity dissected or downplayed in the therapy space led them to interrogate their faith. It created a more subtle, temporary loss of confidence in their faith.

I questioned whether I was a good enough Muslim and whether God thought I was living up to the standard of Islam. (P23)

It also became a battleground for identity with participants describing how they often felt forced to navigate contradictions between their worldview shaped by religion and between the secular framework that underpinned their therapeutic work. It was not just about doubting their religion, it was about doubting their identity, their sense of belonging.

I started feeling that therapy wasn't built for people like me there's a certain kind of people it works for, and I wasn't it. That was a hard pill to swallow. (P8)

### Subtheme 2.1. Religion is my identity, not my mental health

A core problem faced by participants was how Islam was often treated as a problem or a symptom. Participants expressed a clear distinction between their religious identity and their mental health struggles, emphasising that their faith was central to who they were.

It was like they couldn't see me as a person with faith, just as a person with illness. (P12)

The irony of this situation was that they felt therapists were often asking them to look beyond their religion, while simultaneously reducing them to that very identity. This made participants feel they were being seen as a single dimension, despite their complexity as individuals.

My therapist kept asking me if Islam was the problem whether it was getting in the way of me getting better, but Islam gives me a sense of peace that I don't get elsewhere. Why should I have to explain that to my therapist […] I switched off after that, I felt numb as it was another person who just didn't get me. Another person who judges my religion and sees me as extreme. It felt like she was thinking she was saving me from Islam, but Islam is what saved me. (P13)

Participants spoke of trying to explain how Islam shaped their choices and worldview, only for therapists to frame their religious practices in pathological terms.

Every time I attempted to talk about Islam and how it's helping me cope, they would pull the conversation back to my mental health […] she acted like me praying more was a symptom like she only saw me through a pathological lens. (P19)

Participants felt their beliefs were being **medicalised** stripped of their religious meaning and reinterpreted through a Western psychological framework that saw devout practices as problematic. This framing left participants feeling **defenceless**.

It just felt like she was trying to interpret Islam through this clinical lens, which didn't fit with how I viewed Islam […] I get therapists have their models but surely, you don't make people feel their way of living is wrong […] The whole experience was all quite overwhelming. (P14)

There was a sense that Islamic ways of knowing, coping and healing were **not recognised as legitimate** within therapeutic discourse. For participants, this not only diminished the significance of their faith, but it was also emphasised in a way that felt both dismissive and reductive.

I mentioned to her that I feel sometimes God is testing me, and she asked if I often feel like I'm being punished. I had to explain that this isn't paranoia it's literally part of my belief that life is a test. She didn't get it. (P5)

For participants with psychosis, this differentiation between religion and mental health remained clear even during episodes. This distinction was particularly important for participants, as they felt that therapy often blurred these lines, with some therapists assuming that religious experiences or expressions such as praying, mentioning jinn or attributing divine will were part of their illness rather than recognising these as normative, meaningful aspects of their faith. For example, a participant noted that feeling God's presence was mistaken for hallucination, despite them clearly identifying it as part of their faith.

Even when I was having my psychotic episode, I knew Islam wasn't part of the illness. My reality might have been scattered, but my belief in God stayed solid. (P19)

This insight underscores the depth of participants faith, demonstrating how even in times of extreme mental distress, they were able to distinguish between their religious beliefs and their illness. Their ability to differentiate between their religious identity and their mental health condition, even in moments of acute distress, highlights the significance of their faith and the role it plays in their lives.

### Subtheme 2.2. Lack of feeling safe in therapy

This subtheme highlights the pervasive sense of unsafety participants experienced in the therapeutic setting, often linked to their religious identity and the cultural dissonance they felt during sessions. For many, therapy did not provide the emotional security needed to openly discuss their struggles, particularly around issues related to their Islamic identity. The absence of safety in therapy hindered the formation of trust, a crucial element in any therapeutic relationship.

Without the trust, therapy became a superficial exercise in coping, rather than a space to explore my difficulties. (P3)

This restraint was not just about protecting themselves; it was about the very real fear that their most intimate beliefs and values would be misconstrued or dismissed. It highlighted the feelings of vulnerability that many clients experience in therapy.

It's always felt like I've stood out like a sore thumb being a Muslim that wears a hijab and then to be in a space where it's meant to be a safe space and still feel like you're not fitting the therapist criteria. Where do I fit in? (P8)

Several participants reflected that there appeared to be subtle implicit and explicit biases from their therapist, which they perceived as microaggression.

I felt like my therapist had this undertone like, are you oppressed? It's as if I was being assessed through that lens. (P9)

This perceived microaggressions they experienced within therapy, whether intentional or not, reiterated the broader common discourse surrounding Islam. It left some participants feeling stereotyped or unwelcome. These included therapists questioning whether wearing the hijab was a personal choice, or framing fasting during Ramadan as a clinical risk without exploring its spiritual significance.

She asked me if I was forced to wear the hijab. I told her no, it's my choice, but it made me feel like she saw Islam as something oppressive. (P3)

These experiences of linking religious identity to victimhood made participants question the therapists' ability to understand and support them, ultimately harming their sense of autonomy and dignity. For some, this mirrored larger societal experiences of marginalisation, where Islam is often misunderstood or misrepresented.

We are already targeted in the media and by society, I just didn't think it would be the same in therapy too. (P5)

### Theme 3. Feeling compelled to choose between my faith or therapy: ‘I Rather Get No Help for My Mental Health than Someone Making Me Question My Religious Identity.’ (P5)

This theme highlights the intricacies and challenges that occurred when participants' religious identity was not fully acknowledged within therapeutic settings. It implies a conscious choice for some participants to prioritise religious beliefs over therapeutic practices that fail to acknowledge or respect these beliefs. Islam was a non‐negotiable aspect of their identity and one they were not willing to compromise for the sake of therapy.

It was really frustrating. Islam is my whole life like, it's how I view the world. It felt like a big part of my identity was being ignored, which made it harder, I guess, to connect and fully engage in my therapy because what is the point in me opening up. If they don't care about how important religion is to me. Then they don't care about me as a person. You can't separate the two. (P20)

The resolution was achieved through degrees of disengagement from therapy. Some participants disengaged internally, and continued to attend, while others redirected their support‐seeking to Muslim therapists or community/faith‐based alternatives that integrated their faith and offered a more holistic approach towards their mental health. For a small number, disengagement left a sense of hopelessness, reasoning that if therapy could not meet their needs, then perhaps no form of adequate help was available.

I ended up seeking support from a Muslim therapist that my Mum found, he was so much better, I didn't have to explain things to him he just got it. (P21)

The emotional consequences of disengagement were significant. Participants spoke of sadness, loss, anger and diminished trust in the wider mental health system. What united their accounts was a sense of exclusion and being compelled to turn away from therapy to protect themselves.

I left each session feeling helpless, but also angry, angry at myself and angry at my therapist. (P16)

### Subtheme 3.1 flag bearer for Islam, the irony of identity in therapy

Many participants expressed how, as well as disengaging from therapy as a source of personal acceptance and psychological support, they found themselves justifying Islamic religious practices and defending their beliefs in their sessions. This left some participants feeling frustrated, as though in therapy, too, they had to fight to voice the importance and legitimacy of Islam in people's lives.

I have to be representative of Islam in therapy too. I don't just get to be a human. (P11)

For some, this felt like a battle ensuring that Islam was not misinterpreted or judged by therapists. This created a dynamic in which therapy, rather than serving as a safe space became another context where their religious beliefs were questioned.

I felt like I had to keep explaining that my beliefs weren't extreme or dangerous. I was always justifying things why I pray, why I fast like I had to prove Islam wasn't the problem. (P9)

A repeated theme for many of the participants was the need to educate their therapist who either had a lack of knowledge, understanding or viewed Islam and its followers as extreme.

I thought therapy was for me to get help, not to sit there and constantly correct and educate my therapist about Islam. (P24)

I had to teach my therapist the difference between forced marriage and an arranged marriage and how Islam forbids forced marriages. (P4)

Instead of therapy being a space to process their difficulties and address mental health concerns, it became a space in which they were having to defend who they were as a person.

I just want to be a person, a Muslim person, who needs support for their mental health. I should not have to defend my religion. It's part of me and I should not have to defend me. (P13)

The impact of this experience was an overwhelming sense of not belonging, which led to participants feeling emotionally drained and hesitant to bring up Islam in their sessions. The defensiveness extended outside of individual therapy, with some participants being afraid about how Islam was perceived in mental health care.

## DISCUSSION

The present study is the first of its kind to directly explore Muslim clients' experience of therapy when they felt their religion is minimised or excluded. The findings produced three main themes and four subthemes: feeling powerless and unseen when attempting to bring Islam (theme 1), shaking my foundation (theme 2) and feeling compelled to choose between my faith or therapy (theme 3). The themes identified highlight the complex experiences of disempowerment, safety and self‐conception within the therapeutic environment.

The study highlights how Muslims accessing therapy can experience feelings of constraint and powerlessness when their religious perspectives are not actively recognised. This shows the broader difficulties within psychotherapy, which tends to function and prioritise psychological aspects of identity over religious identity (Gunasinghe et al., 2019; Milstein et al., 2010). These findings must be understood within the wider context of being Muslim in the western world, where Islam is often racialised, politicised and pathologised (Mansouri, 2020). Therefore, participants' experiences of feeling marginalised in therapy may reflect the societal patterns in which Islam is perceived as extreme or as incompatible with dominant secular norms (Ahmad, 2020).

Owen et al. (2016) went further and explained how, when participants perceive judgement from their therapist or feel misunderstood, it can lead to feeling overlooked in mental health settings. This mirrors wider literature, which raises concerns about the history of psychology and its link with systems of oppression that have marginalised minoritised groups (Cullen & Walsh, 2020). These findings support calls for a shift towards a more collaborative, culturally responsive framework that validates and integrates clients' religious beliefs as central to their wellbeing.

Fricker's (2007) theory of epistemic injustice helps explain how Muslim clients felt dismissed when religion was minimised or excluded in therapy. Testimonial injustice was reflected in participants feeling discredited or silenced when expressing their religiousness, while hermeneutical injustice captured the lack of a shared framework to make sense of therapy that felt religiously misaligned. This exemplifies the paradoxical nature of the participants' experiences, whereby the system designed to support their mental health is the very system that makes them feel further vulnerable. These findings suggest that the exclusion of religion in therapy is not just a clinical oversight but a form of systemic injustice. Applying this theory highlights the need for therapeutic models that validate clients as credible knowers of their own religious identities.

The study reveals that for many individuals, religion is not only an aspect of their identity but also the primary lens through which they understand both themselves and the world around them. Social identity theory (Turner et al., 1979) facilitates an understanding of the tension participants experienced between their religious identity and the dominant norms of therapy. The therapeutic space threatened a core part of participants' social identity, as it implicitly positioned western secular worldviews as normative. This can reinforce in‐group/out‐group dynamics, leading clients to feel they had to choose between being authentically Muslim or being therapeutically understood. This internal conflict reflects the psychological costs of identity threat and sheds light on why some disengaged or felt unsafe. Conversely, for Muslims whose religious identity may be less central to their self‐concept, the exclusion of religion in therapy might not evoke the same degree of distress or disconnection.

The present findings provide further evidence that a one‐size‐fits‐all approach is inadequate for individuals whose identities are deeply connected to religious beliefs. The biopsychosocial‐spiritual (BPSS) model offers a valuable starting point for considering the spiritual domain integral to psychological health (Engel, 1979). It recognises that mental health is not shaped only by biological, psychological and social factors, but also by an individual's spiritual or religious beliefs, practices and experiences. However, in practice, the spiritual component is often underdeveloped, with therapists lacking confidence, training or theoretical grounding to explore clients' religious worldview (Crossley & Salter, 2005; Islam & Chadwick, 2025). The findings suggest that integrating this domain more intentionally and not as an optional add‐on may help clients feel more seen, validated and safe. For the participants in this study, religion was not merely a cultural marker, but a core aspect of their identity, coping strategies and conceptualisation of distress and healing. Models like the BPSS must be adapted to give religion equal weighting to other parts of identity.

Existing literature on the culturally adapted care in therapy sheds light on how a therapist may distance themselves from client's faith as a self‐protective strategy (Arshad & Falconier, 2019; Bier, 2022; Clorina et al., 2024). In examining why therapists avoid the discussion of religion, particularly in the context of Islam, it could be the fear of unintentionally coming across as Islamophobic (Abu Raiya & Pargament, 2010). However, when therapists sidestep these conversations, they miss out on essential aspects of the client's identity from the therapeutic process. This subject points to a broader challenge within the field of psychological therapies: the need for better training and greater confidence among therapists in addressing religious issues (Tillman et al., 2013). Currently, many therapists rely primarily on their own experiences and learning (Golsworthy & Coyle, 2001). Therapists being equipped with the knowledge and skills to engage in sensitive topics in a respectful and informed manner, the fear of being perceived as ‘Islamophobic’ can be mitigated (Vieten et al., 2013). Therefore, while the fear of ‘cancel culture’ is a valid concern, it is crucial that this fear does not lead to the exclusion of the client's religious identity from the therapeutic process. Progress is happening, with approaches such as Spiritual Integrated Psychology (SIP), Islamic Psychology and multifaith therapies offering more structure and flexible means of integrating religion into the therapeutic process (Captari et al., 2022; Cucchi, 2022; Pargament, 2007).

### Implications for practice

The findings from this study have several implications for therapeutic practice—both within the NHS (where 15 participants received the therapy of interest) and outside. First, mental health services in the UK should strive to increase the representation of Muslim therapists, ensuring they are able to supervise and support therapists working with this population.

Second is the need for clinical training to go beyond ‘cultural competence’ and instead foster ongoing self‐reflection and awareness of unconscious bias. This could include religious literacy and humility as this could help mitigate the risk of disengagement with services (Hassan et al., 2024).

Third, the study highlights the need for a critical examination of how therapy is framed and delivered to Muslim clients. To address this, there needs to be a change in outlook from religion as private to religion as central to identity. For example, early in the therapeutic relationship, the therapist would benefit from open questions to invite the client to talk about their faith. This shift requires supervision and service‐level reflections.

It is also important to explore how group‐based therapies for Muslim service‐users, rather than individual therapy, might influence the psychological processes observed in the study: for example, discovering universality with other group members is a subjectively powerful therapeutic factor for marginalised groups in psychological therapies (Chadwick et al., 2000).

Fourth, participants' decision to disengage from therapy or seek alternative support points to a structural failure of services to retain and support religiously minoritised clients. Therefore, it is important to engage closely with the Muslim community and faith leaders to co‐design interventions and gain deeper insights into the specific religious, cultural and healthcare needs of this population.

Finally, while religion is undeniably important for many clients, it is essential we do not impose a religious framework on those who do not want it. Rather than swinging to either extreme, ignoring religion entirely or overemphasising it, we need to adopt a sensitive, client‐led approach that respects individual preferences. This means that therapists should remain open and curious about clients' religious identities without assuming or prescribing how these should be integrated into therapy.

## LIMITATIONS

The limitations of the study should also be acknowledged. The study was only able to recruit English‐speaking participants that can use the phone and/or computer. Similarly, the recruitment process—the study was advertised online, and the majority of emails to mosques and organisations had no response. Consequently, the study may not represent the Muslim community in the UK who did not have access to participate in this research. The data collection relied on self‐reported experiences, which can be subject to various biases, including recall bias and social desirability bias. Also, due to a lack of time, the transcripts could not be checked over by the participants, which would increase the credibility of the study (Shenton, 2004). Future research could also benefit from focusing on a single therapeutic modality to reduce confounding variables (e.g., therapist skills, specific interventions used and duration of therapy).

It is also crucial to acknowledge various factors that may have impacted the participants' experiences and how they shaped their narratives, as this can have an influence on the themes created from the data. For many Muslims, their view on the intersection of Islam and therapy is intertwined with their personal and communal beliefs (Haque & Keshavarzi, 2014), making their interpretations not only a reflection of individual experience in therapy but also expressions of collective religious and cultural identities. Further to this, participants' narratives will reflect broader past experiences including religious marginalisation within the healthcare system (Hui et al., 2020).

Future research would benefit from understanding therapists' support from supervisors in their ability to navigate conversations around religion in a framework that emphasises a ‘Western hegemony’ (Wood & Patel, 2017). It could also focus on exploring psychological therapists' own experiences of addressing (or not addressing) religion in therapy.

## CONCLUSION

This study adds to the growing body of literature that highlights the importance of providing culturally sensitive mental health care to individuals from diverse backgrounds through adopting a person‐centred approach to therapy rooted in cultural humility, relational safety and pluralism. Ultimately, the study calls for a review of current clinical practice. There is a clear imperative for training providers, mental health services and policymakers to prioritise religious competence, ensuring that Muslim clients and all clients are met with curiosity, respect and understanding in their therapeutic work. This will help foster safety, trust and therapeutic effectiveness for clients whose faith is a key part of their lived experience.

## AUTHOR CONTRIBUTIONS

**Rumena Islam:** Conceptualization; investigation; writing – original draft; methodology; writing – review and editing; formal analysis; project administration; data curation; validation. **Paul Chadwick:** Supervision; writing – review and editing.

## CONFLICT OF INTEREST STATEMENT

No conflicts of interest to declare.

## Supporting information


**Table S1.** Application of reflexive thematic analysis.

## Data Availability

The data that support the findings of this study are available on request from the corresponding author. The data are not publicly available due to privacy or ethical restrictions.
