# Supplementary material for: Therapy without faith: Muslim clients' experience of religious exclusion and minimisation in therapy
Source: Psychol Psychother. 2025 Oct 24;99(1):219–37. doi: 10.1111/papt.70019 (PMC12905511; doi:10.1111/papt.70019)
Supplement: Supplementary file 1 — Table S1. Application of reflexive thematic analysis. [file PAPT-99-219-s001.docx]

**Table 1**
 *Application of Reflexive Thematic Analysis*

| Analysis Stage | Aim of Each Analysis Stage | Application of Analysis Stage and Reflexivity |
| --- | --- | --- |
| 1. Data familiarisation | Immersing in and familiarising with the data to gain an initial understanding. | RI listened to the audio of each interview to ensure that the transcripts were recorded accurately. Transcripts were read and re-read to become immersed with the data, gaining a comprehensive understanding of participants’ perspectives on how religion impacts their mental healthcare experiences. Initial thoughts and observations were recorded as part of RI’s reflexive log. Through this process, RI identified that certain themes resonated with her experiences of therapy, and so actively aimed to engage with all parts of the interviews equally. |
| 2. Generating initial codes | Identifying important data features by manually producing initial codes across the dataset. | Initial codes were systematically applied by RI to segments of the data that were relevant to the research question. This involved identifying recurring patterns, concepts and themes related to the participants’ experience of therapy. RI’s inductive approach meant that she viewed the transcripts with openness and curiosity for codes without pre-existing theories. RI remained open to each participants’ experience and any biases that came up through this process was noted in the reflexive log. Codes identified by RI were reviewed by EV to ensure agreement, leading to minor changes in the codes. |
| 3. Searching for themes | Sorting initial codes into potential themes and sub-themes to capture patterns of meaning within the data. | Initial themes were developed based on their relevance to the research question and were grouped if they shared meanings or it appeared to be a theme. PC’s review of the themes demonstrated that the themes selected initially were too descriptive, functioning more as topic summaries, and required a deeper level of analysis. This prompted a meeting with PC to discuss how the analysis could better capture the experience of the participants. Through a conversation and with prompting questions from PC helped provide a better understanding of how the data was gained. Both RI and PC were able to collaboratively come up with themes that better explained the feelings felt by participants when their faith was excluded or minimised. This prompted RI to extend the analysis further and re-read the transcript to help develop themes that represented underlying meaning. Using the reflexive log to document this process, RI noticed feeling surprised that she was missing depth to her analysis. |
| 4. Reviewing themes | Ensuring themes are consistent across the dataset and refining them into a thematic map. | These themes were reviewed and refined to ensure they accurately reflected the data and provided a coherent account of participants’ experiences. Through this review, RI identified areas where themes overlapped and so this was refined for greater precision. Meetings were also held with GA to support this process. RI then developed the analysis with the themes identified. Reflexivity showed some discomfort in moving away from the expected themes, but helped stay aware of potential assumptions from previous readings of related literature. The review helped form a thematic map creating three themes, with the first and third theme containing one subtheme and the second theme containing two subthemes. |
| 5. Defining and naming themes | Identifying essence of each theme and finalising their names to capture their meaning concisely. | Clear definitions were established for each theme and integrated into a coherent narrative that illustrated how religion shapes the mental healthcare experiences of Muslim clients. RI ensured that each theme had a clear central concept and chose names that accurately conveyed each theme. Final refinements were made, and the thematic map was reviewed. Reflexive practice highlighted a need to balance analytical insight with participant language for authentic representation. |
| 6. Producing the report | Writing up the final report, including both the analytic narrative and relevant data extracts to illustrate key points. | RI selected key quotes that best represented each theme, whilst trying to be mindful of not over-representing certain ideas. There was a pull at times to include excessive number of quotes that conveyed the same message due to worries that interpretation of the points might reflect researcher bias. Therefore, GA and RI worked together to select extracts to convey the analytical claim being made. Feedback from the supervisor PC supported RI to make refinements to the final report, to ensure it aligned with the research question and provided a narrative coherence of the findings. |
